# A high-throughput, whole cell assay to identify compounds active against carbapenem-resistant *Klebsiella pneumoniae*

**DOI:** 10.1371/journal.pone.0209389

**Published:** 2018-12-21

**Authors:** Julie Liao, George Xu, Emily E. Mevers, Jon Clardy, Paula I. Watnick

**Affiliations:** 1 Division of Infectious Diseases, Boston Children’s Hospital/Harvard Medical School, Boston, Massachusetts, United States of America; 2 Department of Biological Chemistry and Molecular Pharmacology, Harvard Medical School, Boston, Massachusetts, United States of America; 3 Department of Microbiology and Immunobiology, Harvard Medical School, Boston, Massachusetts, United States of America; University of Georgia, UNITED STATES

## Abstract

Enteric Gram-negative rods (GNR), which are frequent causes of community-acquired and nosocomial infections, are increasingly resistant to the antibiotics in our current armamentarium. One solution to this medical dilemma is the development of novel classes of antimicrobial compounds. Here we report the development of a robust, whole cell-based, high-throughput metabolic assay that detects compounds with activity against carbapenem-resistant *Klebsiella pneumoniae*. We have used this assay to screen approximately 8,000 fungal extracts and 50,000 synthetic compounds with the goal of identifying extracts and compounds active against a highly resistant strain of *Klebsiella pneumoniae*. The primary screen identified 43 active fungal extracts and 144 active synthetic compounds. Patulin, a known fungal metabolite and inhibitor of bacterial quorum sensing and alanine racemase, was identified as the active component in the most potent fungal extracts. We did not study patulin further due to previously published evidence of toxicity. Three synthetic compounds termed O06, C17, and N08 were chosen for further study. Compound O06 did not have significant antibacterial activity but rather interfered with sugar metabolism, while compound C17 had only moderate activity against GNRs. Compound N08 was active against several resistant GNRs and showed minimal toxicity to mammalian cells. Preliminary studies suggested that it interferes with protein expression. However, its direct application may be limited by susceptibility to efflux and a tendency to form aggregates in aqueous media. Rapid screening of 58,000 test samples with identification of several compounds that act on CR-*K*. *pneumoniae* demonstrates the utility of this screen for the discovery of drugs active against this highly resistant GNR.

## Introduction

There is an urgent need for new antimicrobials to combat the inexorable rise of multi-drug resistant bacteria as the options for treatment of infections caused by carbapenem-resistant Gram-negative bacteria and, in particular, the carbapenem-resistant *Enterobacteriaceae* are limited [1]. For this reason, the Centers for Disease Control and Prevention as well as the World Health Organization have designated carbapenem-resistant *Enterobacteriaceae* a threat that merits the highest priority for research and development of new antibiotics [2]. The increasing incidence of infections caused by carbapenem-resistant (CR) *Klebsiella pneumoniae*, which belong to the *Enterobacteriaceae* family, is particularly concerning because a recent meta-analysis of reports published between 1999 and 2015 found that the worldwide mortality of patients infected by CR *K*. *pneumoniae* could be as high as 50% [3].

Gram-negative bacteria are intrinsically more resistant to antimicrobial compounds than Gram-positive organisms due to (i) the requirement for penetration of both the negatively charged outer and lipophilic inner membranes, (ii) stringent transcriptional and post-transcriptional control of porin expression in the outer membrane, and (iii) expression of multi-drug efflux pumps [4–6]. As a result of these barriers to the entry of small molecules into cells, many bioactive compounds discovered in cell-free, targeted screens do not retain their activity in whole cell assays [7]. For this reason, whole cell screens can be quite useful in identifying antimicrobials active against Gram-negative organisms.

Here, we describe the use of a high-throughput, sensitive whole cell metabolic screen to identify compounds in fungal extracts and synthetic compound libraries with antimicrobial activity against carbapenem-resistant *Klebsiella pneumoniae*. Patulin, a known fungal metabolite that has been shown to inhibit bacterial quorum sensing and alanine racemase, was identified as the active component in the most potent fungal extracts [8]. We did not study patulin further due to its known toxicity [9, 10]. Three synthetic compounds termed O06, C17, and N08 were chosen for further study. Compound N08 was quite active against several resistant GNRs as well as methicillin-resistant *Staphylococcus aureus* (MRSA) and showed minimal toxicity to mammalian cells. However, its utility may be limited by its susceptibility to bacterial efflux and tendency to form aggregates in aqueous media. Compound C17, which was the least toxic to mammalian cells, had only moderate activity against GNRs, and compound O06, which was the most toxic to human cells, had limited antibacterial activity but inhibited sugar utilization. While the antibacterial compounds identified here have characteristics that limit their development as therapeutics, these results establish the utility of our screen in identifying compounds active against carbapenem-resistant *Klebsiella pneumoniae*.

## Materials and methods

### Strains and media

Carbapenem-resistant *Klebsiella pneumoniae* strain ATCC BAA-1705 was used for the HTS. A carbapenem-susceptible clinical strain of *Klebsiella pneumoniae* (Kp233) and a clinical isolate of *Acinetobacter baumanii* available in our laboratory were used for antimicrobial susceptibility studies. *Pseudomonas aeruginosa* PAO1 was generously provided by the laboratory of Dr. Simon Dove. MRSA and Group A streptococcus (GAS) were used in tests of minimum inhibitory concentrations (MIC). These isolates were generous gifts from the laboratory of Dr. Michael Wessels.

*K*. *pneumoniae*, *P*. *aeruginosa* and *Escherichia coli* strains were cultured in Luria-Bertani broth (LB, BD Difco). *Acinetobacter baumanii* and MRSA were grown in tryptic soy broth (TSB, Becton-Dickinson), and GAS was grown in Todd-Hewitt broth supplemented with 0.5% yeast extract (Becton-Dickinson). Cation-adjusted Mueller Hinton broth with 10 mg/L Mg^2+^ and 20 mg/L Ca^2+^ (Becton-Dickinson, CAMHB) was used in MIC assays. CAMHB supplemented with 2.5% lysed horse blood was used for GAS. Where indicated, 40 μg/mL of the efflux pump inhibitor phenylalanine-arginine beta-naphthylamide (PaβN, Sigma) was added to the medium to inhibit the multi-drug efflux pumps of the Gram-negative bacterial species under study [11]. Where required, carbenicillin (Sigma) was added at 150 μg/mL for plasmid retention. Frozen stocks were maintained at -80°C in 15% glycerol.

### Fungal extract and chemical compound libraries

A library of desiccated fungal extracts was generously supplied by Dr. David Newman and Carol Haggerty at the Natural Products Branch of the National Cancer Institute and resuspended at a concentration of 15 mg/ml in dimethyl sulfoxide (DMSO). A chemical library comprised of compounds from the Targeted Diversity Library at ChemDiv and purchased and maintained by the Institute of Chemistry and Chemical Biology-Longwood (ICCB-Longwood) at Harvard Medical School was also screened. The Targeted Diversity Library consists of approximately 50,000 drug-like compounds built around 2,500 diverse chemical scaffolds. All compounds in this library were resuspended in DMSO yielding a concentration of 5 mg/mL and stored at -80°C.

### High-throughput screen (HTS) based on sugar fermentation

Libraries were screened using a HTS that detects inhibitors of mannose fermentation by strain *K*. *pneumonia* BAA-1705 in pH-DM medium [12]. In preparation for screening, a small amount of a frozen glycerol stock of *K*. *pneumoniae* was spread on an LB agar plate and incubated overnight at 37 °C. A loopful of bacteria was collected, washed three times in normal saline (NS), and resuspended in NS to yield an optical density at 600 nm (OD_600_) of 0.016. The HTS was performed in a defined medium containing mannose (pH-DM, S1 Table) and supplemented with thymol blue (0.006% w/v) and bromothymol blue (0.006% w/v). The pH was adjusted to 7.6 by adding sodium hydroxide to a final concentration of 1 mM. All reagents for the defined media were obtained from Sigma-Aldrich.

Wells of a 384-well plate were filled with 30 μL of pH-DM using a Matrix WellMate liquid handler (ThermoScientific). 100 nL of each test extract or compound dissolved in DMSO was added at the ICCB-Longwood facility by pin-transfer using a custom-built Epson robot. Columns 23 and 24 of each plate were set aside for controls. Column 23 received no compound and served as the negative control. Column 24 contained pH-DM supplemented with 50 μg/mL of gentamicin and served as the positive control. Plates were transported back to our laboratory where 10 μL of the bacterial suspension was added to yield a final extract concentration of 37.5 μg/ml or a final compound concentration of 12.5 μg/ml. Plates were covered and incubated in stacks of 4 at 27 °C. Each plate was screened in duplicate. To reduce evaporation from wells on the plate perimeter, the incubator was humidified with containers of deionized water. The A_615_ was measured on an Infinite 200 spectrophotometer (Tecan) at the start of the incubation (0 hr), after 7 hours (7 hr), and after 22 hours (22 hr). The results were plotted in the Dotmatics Vortex software suite to visualize heat map and scatterplot graphs.

### Identification of patulin in active fractions of fungal extract F19

An aliquot of F19, an active fungal crude extract, was purified using reverse-phase high pressure liquid chromatography (RP-HPLC) with a Luna C18(2) 5μ 10x250 mm column (Phenomenex) under the following gradient: holding 10% ACN + 0.1% formic acid (FA)/90% H_2_O + 0.1% FA for 2 min then gradient to 100% ACN + 0.1% FA over 20 min flowing at 3 mL/min to generate five fractions (A-E). Only fraction B exhibited activity and dereplication using high resolution mass spectrometry (HRMS) revealed patulin as the active component. All additional active fractions were screened for the presence of patulin using a low-resolution LCMS equipped with a Luna C18(2) 5μ 4.6x100 mm column (Phenomenex) and run using the following gradient system: holding 10% H_2_O/ACN + 0.1% formic acid (FA) for 2 min then gradient to 100% ACN + 0.1% FA over 17 min flowing at 0.7 mL/min. All 4 extracts analyzed with strong activity were confirmed to contain patulin.

### Commercial supply of compound hits

Patulin was purchased from Cayman chemicals. Three prioritized synthetic compounds were also purchased. Compound O06 was purchased from ChemDiv under the catalog number 8019–0105. Compounds C17 and N08 were purchased from Vitas-M Laboratory under catalog numbers STK871080 and STK250799, respectively.

### Dose-response assays

Two-fold serial dilutions of chemical compounds in pH-DM or LB broth as noted were prepared in the wells of a 384-well plate. The compounds ranged in concentration from 0.78 μg/mL to 50 μg/mL. Carbapenem-resistant *K*. *pneumoniae* strain BAA-1705 was prepared as described for the HTS, and 10 μL of the bacterial preparation was added to 30 μL of the compound suspensions. Mannose fermentation was measured spectrophotometrically as described for the HTS in wells filled with pH-DM. Viable cell counts were measured in wells filled with LB broth.

### Alamar blue assay for cytotoxicity

HeLa S3 cells were cultured in Dulbecco’s modified Eagle medium supplemented with 10% fetal bovine serum, 100 U/mL penicillin, and 100 μg/mL streptomycin. Each well of a 96-well microtiter plate (Nunclon Delta Surface, Thermo Scientific) was seeded with 10,000 cells in 100 μL of culture medium. Cells were incubated for 18 hr in a humidified incubator at 37°C with 5% CO_2_ to allow attachment. Compounds were added to the adherent cells to yield a final concentration of 13 and 107 μg/mL. Positive control wells contained 0.1% Triton X-100, carrier control wells contained DMSO, and negative control wells contained medium and cells without DMSO. After addition of compounds, 0.1% Triton, or DMSO, plates were returned to the incubator. Cell viability was measured by resazurin reduction. Alamar Blue reagent (Invitrogen) was added to the cells after a 1.5 hr incubation with the test compounds. Cells were incubated for 20 hr, and then fluorescence was measured using the Infinite 200 (Tecan) with excitation and emission wavelengths of 550 nm and 590 nm, respectively. To calculate viability, the average fluorescence value of Triton-treated positive control wells was subtracted from that of untreated wells or wells containing test compounds. These values were then divided by the average fluorescence of untreated wells to give cell viability as a percentage of the negative control.

### Minimum inhibitory concentrations (MIC)

To measure the MIC, two-fold dilutions of compounds were prepared in Mueller Hinton broth, resulting in concentrations ranging from 1.5 μg/mL to 192 μg/mL. Stationary phase bacteria were inoculated into CAMHB with or without the efflux pump inhibitor PAβN to yield a final OD_600_ of 0.1 and then diluted 100-fold, resulting in a final cell density of approximately 1 x 10^5^ CFU/mL. Fifty μL of the bacterial suspension was added to 50 μL of each compound-containing solution, resulting in a total volume/well of 100 μl. The final concentration of compounds ranged from 0.75 μg/mL to 96 μg/mL, and each well contained approximately 5 x 10^4^ CFU of the indicated bacterium. Compound N08 may not have been fully solubilized at the highest concentrations. Gentamicin ranging in concentration from 0.78 μg/mL to 100 μg/mL was included as a positive control in MIC assays. Levofloxacin and chloramphenicol are known substrates of the multi-drug efflux pumps of *P*. *aeruginosa* and *K*. *pneumoniae*, respectively [13–15]. Therefore, for assays performed with PaβN, levofloxacin concentrations between 3.9 ng/mL and 0.5 μg/mL and chloramphenicol concentrations between 9.8 μg/mL and 1.25 mg/mL were included as positive controls. Plates were covered and incubated for 18–20 hr at 37°C. Bacterial growth was visible as biomass collected at the bottom of the wells. The MIC reported is the lowest compound concentration that visually inhibited bacterial growth.

### Compound impact on bacterial viability

Compound stocks maintained in DMSO were diluted in LB to achieve final compound concentrations of 100 μg/mL, and PAβN was used at 40 μg/mL. One hundred μL of each compound preparation was dispensed into the wells of a microtiter plate. Negative (untreated) wells contained LB with or without PAβN only.

#### Stationary phase cell viability

A glycerol stock of the carbapenem-susceptible clinical strain of *K*. *pneumoniae* (Kp233) was inoculated into 1.5 mL of LB and incubated overnight (approximately 18 hr) at 37°C with shaking. Cells were washed twice with NS and then inoculated into the wells of a microtiter plate prepared as described above to achieve a final cell density of 1 x 10^6^ CFU/ml. Cells were incubated statically at 37°C for 4 hr, and viable counts were determined by plating.

#### Exponential phase cell viability

Overnight cultures of Kp233 were diluted 1:5,000 into 50 mL of LB broth and incubated at 37°C with shaking at 200 rpm. Optical density was measured every 30 min until cells reached exponential phase (OD = 0.4, 2 x 10^8^ CFU/mL). These cells were also washed twice with NS and inoculated into the wells of a microtiter plate prepared as described above. Cells were incubated statically at 37°C for 4 hr, and viable counts were determined by plating.

### Morphometric analysis

Carbapenem-susceptible Kp233 were treated as described for stationary phase viability assays, with the exception that negative control wells contained no DMSO and no antimicrobials. Ampicillin (150 μg/mL) was used as a positive control. Cells were incubated statically at 37°C for 4 hr. Thirty μL from each well was transferred into a 24-well plate filled with 300 μL of LB broth. Images were captured with a 40x magnification lens using a Nikon Eclipse TE2000-E microscope. Morphometric analysis was carried out using IPLab 4.0 software.

### GFP fluorescence assays

To assess protein synthesis, plasmid pJBA110, encoding a variant green fluorescent protein (GFP) with a half-life of 40 min, was transformed into Kp233 to yield PW2127. Cells from the glycerol stock were spread on LB agar containing 150 μg/mL of carbenicillin and incubated at 37°C for 18 hr. Cells were collected and washed twice with sterile NS. Each well of a 384-well clear-bottom plate with black walls was filled with LB containing 4 x 10^5^ CFU of Kp233/pJBA110 and compounds at 1, 10, or 100 μg/mL. Fluorescence was measured every 30 min with excitation and emission wavelengths of 480 and 515 nm, respectively.

## Results

### Implementation and validation of a high-throughput screen for compounds with activity against carbapenem-resistant *Klebsiella pneumoniae*

**Fig 1:**
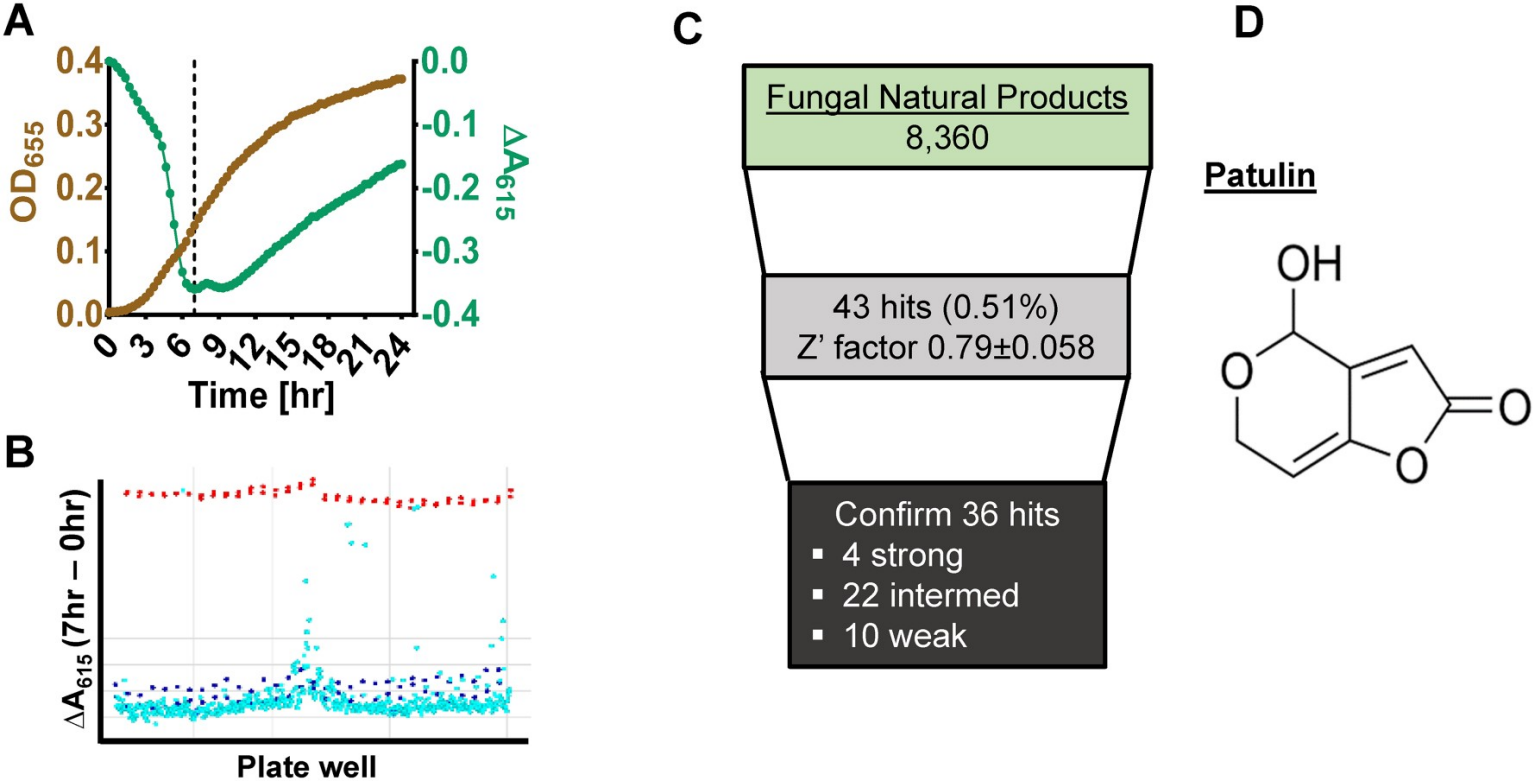
A high-throughput screen based on bacterial fermentation identifies fungal extracts active against multidrug resistant *Klebsiella pneumoniae*. **The antibacterial natural product is patulin. A**, Correlation between bacterial growth measured by optical density (OD_655_) and color change of the medium as a result of bacterial fermentation of mannose (ΔA_615_). The nadir of the color change occurs at early- to mid-exponential growth and is indicated by the dashed line. **B**, Representative data from two screen plates with ΔA_615_ (y-axis) plotted against well location (x-axis). Red circles indicate the measurement using gentamicin as a positive control. Dark blue circles indicate negative controls (medium with no antimicrobials). Light blue circles indicate sample wells that contain unknown compounds. **C**, Summary of the outcomes from the fungal extract library screened. Patulin was a component of all strong hits. **D**, The chemical structure of patulin.

We previously screened natural extracts for activity against *V*. *cholerae* using an assay that measures sucrose fermentation and found it to be extremely sensitive and robust [16]. This assay depends upon the use of a defined reporter medium containing essential amino acids, the pH indicators bromothymol blue and thymol blue, and a fermentable sugar. In this medium, bacterial fermentation results in medium acidification leading to protonation of both pH indicators and a color change from green to yellow. This color change is easily detected spectrophotometrically as a decrease in the absorbance at 615 nm (A_615_). We optimized this assay for *K*. *pneumoniae* by using mannose rather than sucrose fermentation as the indicator of metabolic activity. *K*. *pneumoniae* mannose metabolism resulted in a decrease in A_615_ after incubation at room temperature for 3–5 hr. The A_615_ nadir was reached after approximately 7 hours, at the mid-exponential phase of growth, and had recovered considerably after 20 hours (Fig 1A). Therefore, in our high-throughput screen (HTS), A_615_ measurements were taken at 7 and 20 or 22 hours.

To validate the screen, we first tested two plates from the Biomol 4 federal drug administration-approved known bioactive compound library available at the ICCB-Longwood Screening Facility. The Z’ factor, a coefficient that reflects the separation of the positive and negative controls, was calculated using the following equation: Z’ = 1-3(σ_p_+σ_n_)/│μ_p_-μ_n_│, where σ_p_ and σ_n_ are the standard deviations and μ_p_ and μ_n_ are the means of the positive and negative controls, respectively [17]. A Z’ factor of 0.7 or higher indicates a good to excellent assay. The Z’ factor for these test plates was 0.750±0.068 (range 0.676–0.821). Our pilot screen identified several known antibiotics as well as DNA-damaging antineoplastic agents with activity against *K*. *pneumoniae* (S2 Table). This further validated our assay.

### High-throughput screen of fungal extracts

We utilized our assay in a HTS against CR *K*. *pneumoniae* using a library of 8,179 fungal organic extracts supplied by the Natural Products Branch at the National Cancer Institute and selected based on previous evidence of antibacterial activity against *E*. *coli* [12]. For quality control, Z’ factors were calculated using controls from replicate plates. Positive and negative controls were generally well-segregated (Fig 1B), with the Z’ factors for replicate plates ranging from 0.73 to 0.91. Plates with unacceptable Z’ factors were repeated. For the fungal screen, strong hits were defined as those giving rise to measurements that fell within three standard deviations of the gentamicin positive control at both 7 and 20 hours. Medium hits fell just outside three standard deviations of the positive control at 7 hours but joined the negative control at 20 hours. Weak hits fell close to the negative control but not within three standard deviations of this measurement. We identified 43 fungal extracts with antibacterial activity in the primary screen (Fig 1C). Of these, 36, including 4 strong hits, were confirmed in cherry pick assays. None of these extracts was found to be toxic to cultured HeLa cells (S1 Fig).

One of these, extract F19, was fractionated. The propensity of each fraction to inhibition fermentation and growth is shown in S2 Fig.

Antibacterial activity was localized to fraction B, and the predominant component of this fraction was patulin (Fig 1D). We subsequently demonstrated that all strong hits also contained patulin. This finding likely reflects a lack of diversity in our natural product library along with the highly resistant nature of the *K*. *pneumoniae* strain under study. The patulin MIC for CR and CS-*K*. *pneumoniae* in rich medium was 18.75 and 9.38 μg/ml, respectively (Table 1). In defined medium, the MIC for both *K*. *pneumoniae* strains was 9.38 μg/ml.

**Table 1:** Minimum inhibitory concentrations measured for patulin (μg/ml).

Bacterial strain	LB broth	Defined medium
CR-*K*. *pneumoniae* (ATCC BAA-1705)	18.75	9.38
*K*. *pneumoniae* Kp233^a^	9.38	9.38

^a^ A carbapenem-susceptible clinical strain of *K*. *pneumoniae*

Patulin has not previously been shown to inhibit the growth of CR *K*. *pneumoniae*. However, it is a secondary fungal metabolite with known antibacterial activity and toxicity [18, 19]. While we did not pursue patulin further, this established proof of concept.

### High-throughput screen of chemical compounds

**Fig 2:**
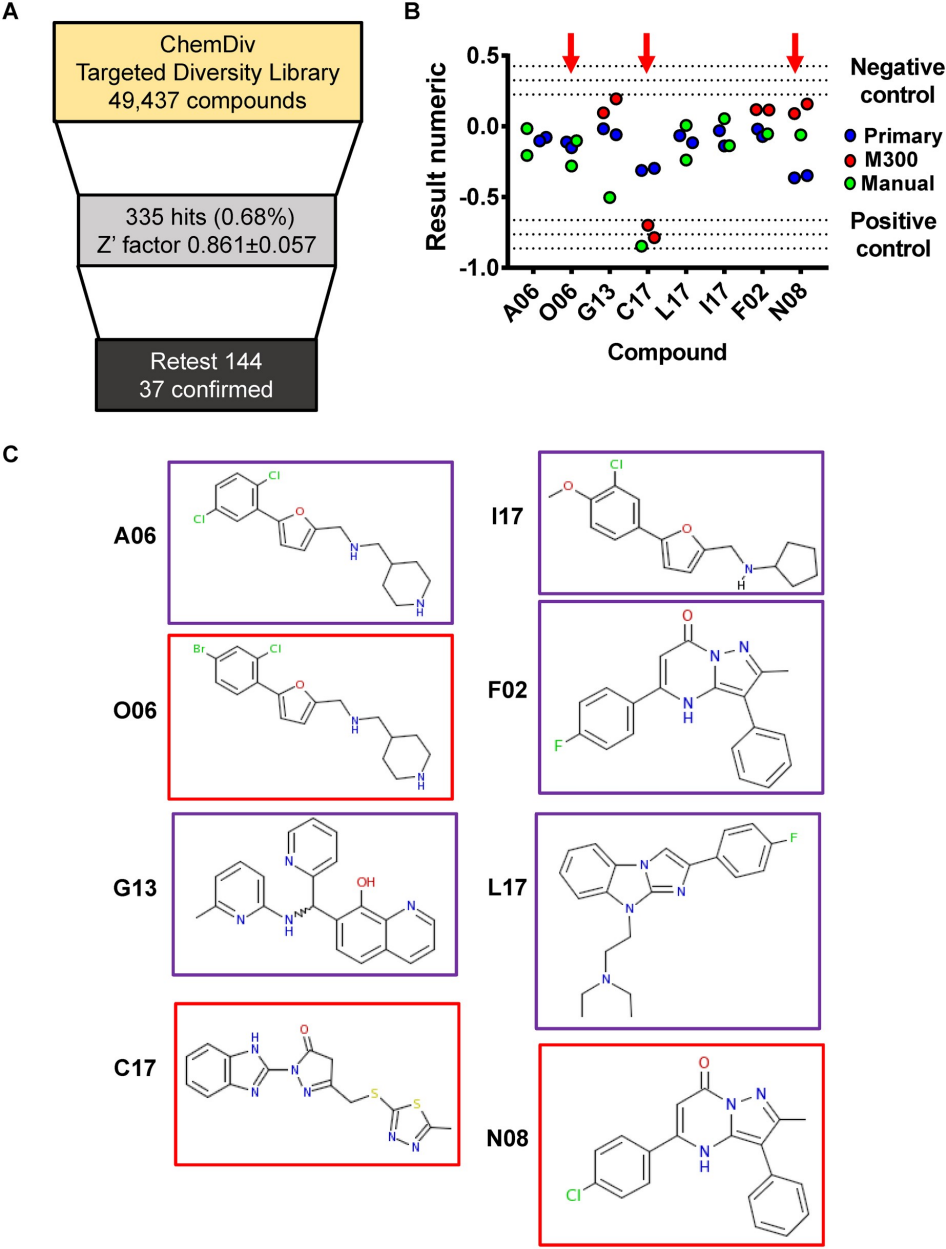
A high-throughput screen based on bacterial fermentation identifies compounds active against multidrug resistant Klebsiella pneumoniae. **A**, Summary of the outcomes from the chemical library screen. **B**, Results numeric of prioritized compounds in the cherry-pick assay. Individual values are shown for primary and cherry pick assays. pH-DM supplemented with 50 μg/mL of gentamicin was used as a positive control, while no compound was added in the negative control. Central dotted lines indicate the average values of the positive and negative controls, while upper and lower dotted lines indicate +/- three standard deviations, respectively. **C**, Structures of top compounds. Compounds selected for further investigation are outlined in red.

Given the uncertainties intrinsic to natural extracts screens, we moved to a screen of chemical compounds from the ChemDiv Targeted Diversity Library. This library consists of small molecules with drug-like properties designed to inhibit known cellular targets including ion channels and enzymes such as kinases and proteases. Compounds from this collection were used at a final concentration of 12.5 μg/ml, and each compound was tested in duplicate. In the primary chemical screen, compounds were considered hits if the well ΔA_615_, defined as the difference in absorbance at 615 nm between hours 0 and 7 of the assay, exceeded the mean ΔA_615_ of the negative control by more than 3 standard deviations. This calculation was referred to as the result numeric. Detailed results of the primary HTS of the ChemDiv library are summarized in Fig 2A.

We re-tested 144 cherry-picked compounds. We initially used an HP D300 digital dispenser with T8 cassettes to generate dose-response curves for 32 compounds, including the 28 strongest hits from the primary screen. However, only one hit, subsequently designated C17, showed significant activity when dispensed by the HP D300 (Fig 2B). We hypothesized that many of our cherry pick compounds had formed small aggregates that obstructed the nozzles of the HP D300 leading to little or no compound release. We, therefore, reverted to dispensing these compounds manually in a 200 nl volume. Using this approach, 37 compounds, amounting to 25.6% of those tested, reached the result numeric threshold set for compound hits. The results numeric in the primary and cherry-pick screens for the eight most active compounds are shown in Fig 2B, and the structures of these compounds are shown in Fig 2C.

### Correlation of fermentation and growth inhibition

**Fig 3:**
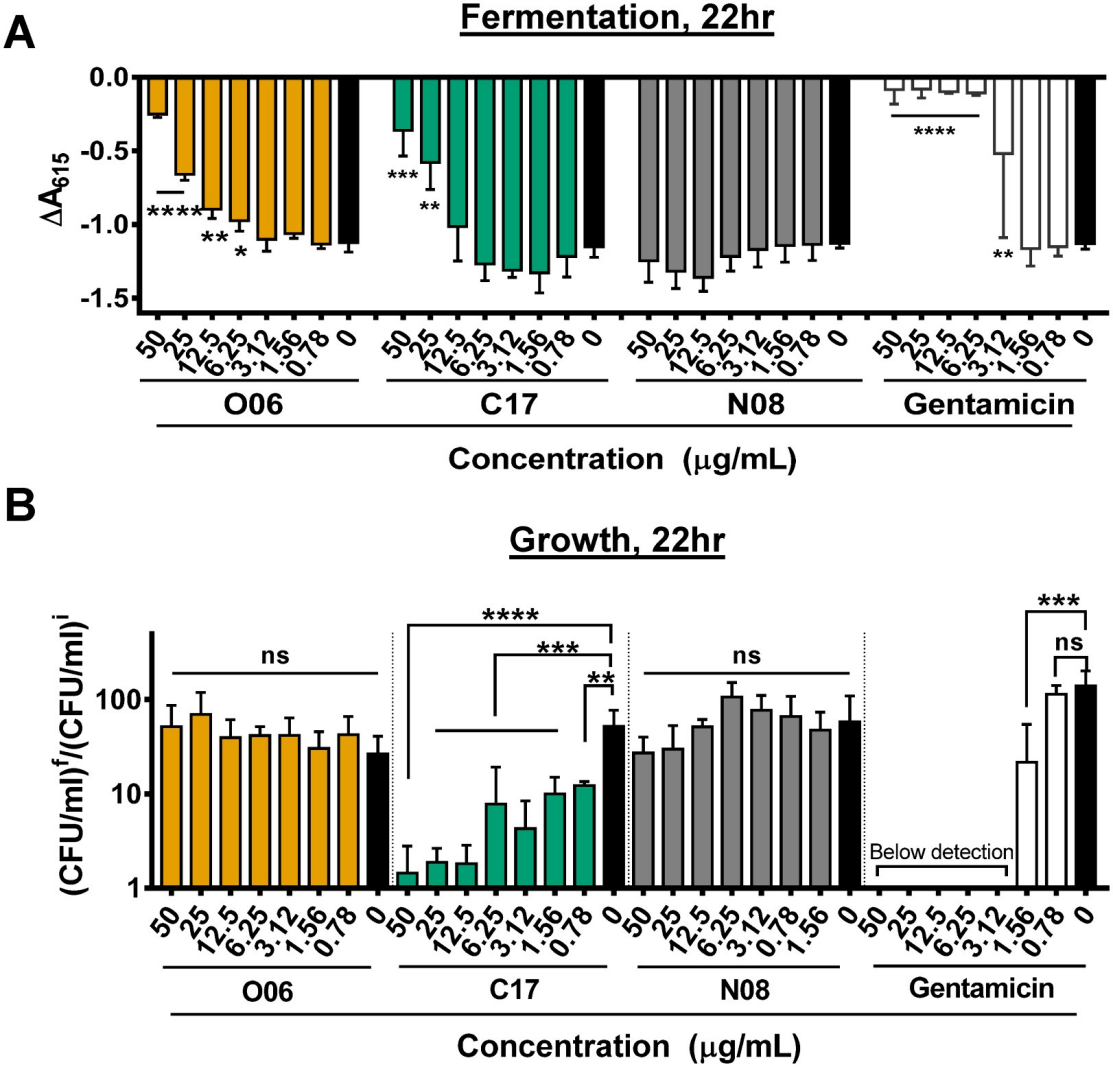
CR-*K*. *pneumoniae* fermentation and growth inhibition by three synthetic compounds identified in the high-throughput screen. Dose-dependent inhibition of **A**, mannose fermentation in DM-pH and **B**, growth represented by CFU/ml after 22 hr of culture in LB broth (CFU/ml)^f^ divided by the CFU/ml at time 0 (CFU/ml)^i^. * *p* ≤ 0.05, ** *p* ≤ 0.01, *** *p* ≤ 0.001, **** *p* ≤ 0.0001, ns *p* > 0.05 using ordinary one-way ANOVA followed by Dunnett’s multiple comparison test. The mean of three independent replicates is shown. Error bars represent the standard deviation. Gentamicin was included as a positive control.

Further investigation of these compounds required the purchase of additional material from commercial vendors. We first established the ability of these new compound preparations to inhibit fermentation. As shown in Fig 3A and S3 Fig, only compounds O06 and C17 inhibited fermentation at 22 hours at concentrations similar to those used in the primary and cherry-pick screens. One possibility is that the other six compounds aggregated at higher concentrations or were not stable over the course of the experiment. Compound C17, which showed activity in the cherry-pick screen regardless of the method by which it was dispensed, was the only compound that inhibited growth of CR *K*. *pneumoniae* at the concentrations tested (Fig 3B).

### Toxicity to human cells

**Fig 4:**
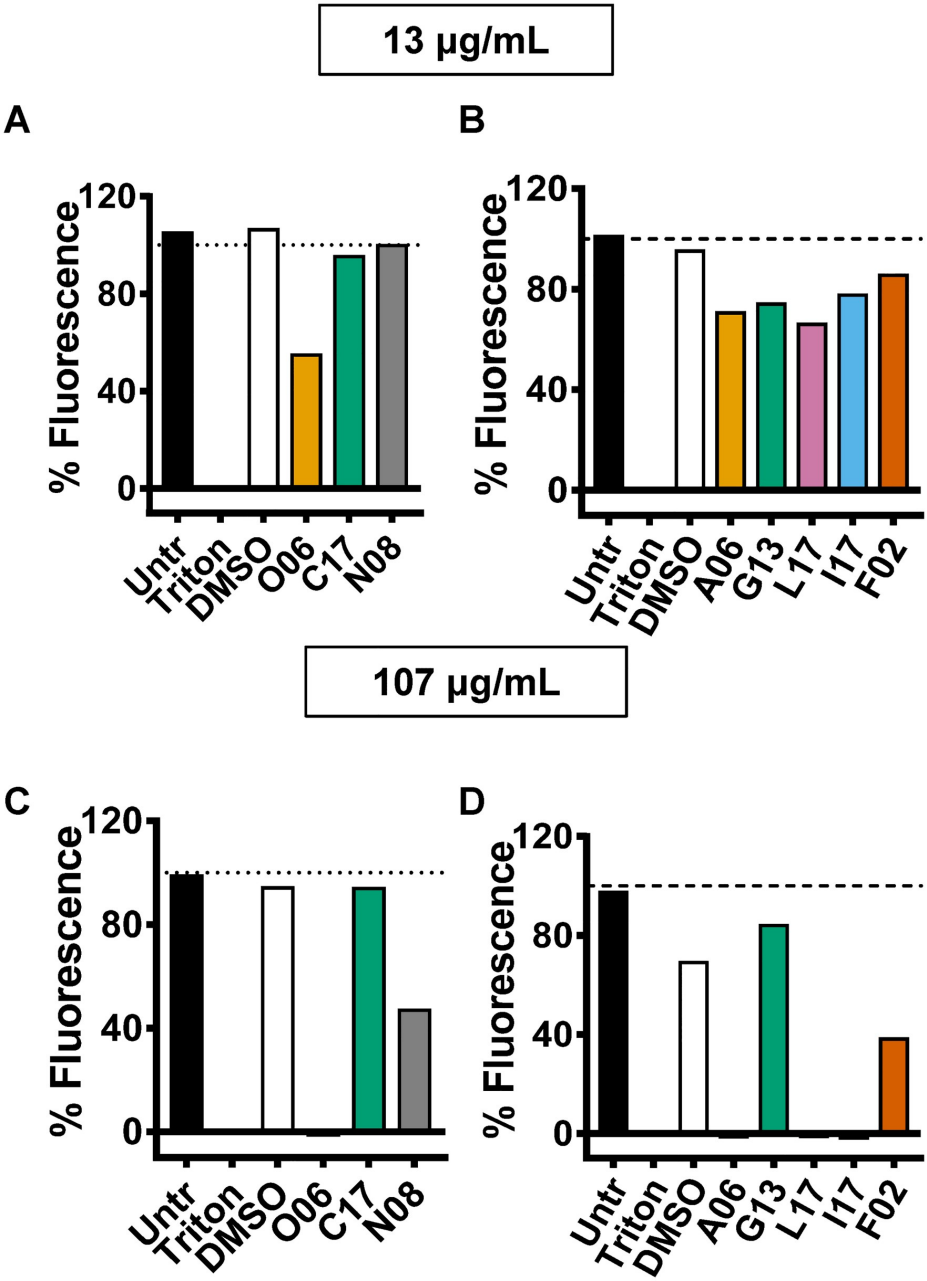
Toxicity of compounds for mammalian cells. HeLa S3 cells were incubated in media containing either compounds or vehicle for 20 hr. Viability was measured by resazurin reduction and expressed as the percent fluorescence compared to untreated cells at (A and B) 13 μg/mL and (C and D) 107 μg/mL. Cells incubated with medium containing DMSO and 0.1% Triton X-100 were included as negative and positive controls, respectively. Untr: untreated. Dotted horizontal line indicates 100% viability.

As a preliminary test of the toxicity of these compounds to mammals, we incubated the eight compounds at concentrations of 13 and 107 μg/mL with HeLa S3 cells for 20 hours in the presence of resazurin. At this point, the fluorescence at 585 nm was measured. The reduction of resazurin to resorufin can be monitored by fluorescence at 585 nm and is indicative of aerobic respiration and, therefore, cell viability. Untreated cells and cells treated with the surfactant Triton X-100 served as controls. As shown in Fig 4, compound C17 and G13 were not toxic at either concentration. Compounds N08 and F02 were only moderately toxic at the higher concentration, while other compounds were quite toxic at the higher concentration. After considering activity in the primary and secondary screens and the extent of toxicity to human cells, we focused on compounds O06, C17, and N08.

### Spectrum of activity of prioritized compounds

To determine the spectrum of activity of compounds O06, C17, and N08, we measured the MIC of each compound against the multi-drug-resistant Gram-negative rods (GNR) *Acinetobacter baumanii*, *Pseudomonas aeruginosa*, and CR *K*. *pneumoniae*, susceptible GNR strains including two uropathogenic *E*. *coli* (UPEC) strains and one *K*. *pneumoniae* strain, and the Gram-positive cocci (GPC) MRSA and GAS (Table 2). The activity of compounds O06 and C17 against the GPCs and GNRs tested was comparable, suggesting that the drug efflux specific to GNRs may not be a significant component of resistance. In contrast, while the GNRs were relatively resistant to compound N08, MRSA was quite sensitive. We hypothesized that efflux might be an important mechanism of resistance to compound N08. To test this, we used the efflux pump inhibitor phenylalanine-arginine β-naphthylamide (PAβN) [13, 20, 21]. To confirm the activity of this pump inhibitor in our strains of *P*. *aeruginosa* and *K*. *pneumoniae*, we first showed that the MICs of levofloxacin and chloramphenicol were reduced 16- and 32-fold in *P*. *aeruginosa* and *K*. *pneumoniae*, respectively (Table 2). We then combined PAβN with our compounds and again tested bacterial susceptibility. As predicted, addition of PAβN minimally decreased the resistant GNR MIC of compounds O06 and C17. In contrast, addition of PAβN reduced the MIC of N08 against resistant GNR by 1–2 logs (Table 2), confirming our hypothesis that efflux is a major mechanism of GNR resistance.

**Table 2:** Minimum inhibitory concentration (MIC) of compounds O06, C17, and N08 against panel of drug resistant Gram-negative and Gram-positive bacteria. Addition of the efflux inhibitor phenylalanine-arginine β-naphthylamide (PAβN) improves the MIC for Gram negative organisms. Cm: chloramphenicol. Lev: levofloxacin.

PAβN (40μg/mL)	Compound (μg/mL)									
	O06		C17		N08		Cm		Lev	
	-	+	-	+	-	+	-	+	-	+
**Multi-drug resistant bacterial strains**										
*A*. *baumanii*	48	24	>96	24	>96	1.5	---	---	---	---
*P*. *aeruginosa* PAO1	>96	24	>96	48	>96	12	---	---	0.25	0.015
CR-*K*. *pneumoniae* (ATCC BAA-1705)	96	96	96	48	>96	6	1250	39	---	---
MRSA	48	N/A^a^	24	N/A^a^	<0.75	N/A^a^	---	---	---	---
**Susceptible bacterial strains**										
*K*. *pneumoniae* Kp233^b^	96	---	48	---	>96	---	<9	---	0.031	---
*E*. *coli* urine isolate Ec235^c^	96	---	96	---	>96	---	<9	---	0.015	---
*E*. *coli* urine isolate Ec236^c^	96	---	96	---	>96	---	<9	---	0.015	---
Group A streptococcus	48	N/A^a^	96	N/A^a^	>96	N/A^a^	---	---	---	---

^a^ Combination of compound with PAβN was not tested in Gram-positive organisms because they do not express resistance-nodulation-division (RND) class of multidrug efflux pumps targeted by PAβN

^b^ A carbapenem-susceptible clinical strain of *K*. *pneumoniae*

^c^ Antibiotic-sensitive clinical isolates of uropathogenic *E*. *coli*

### Compound impact on bacterial viability

**Fig 5:**
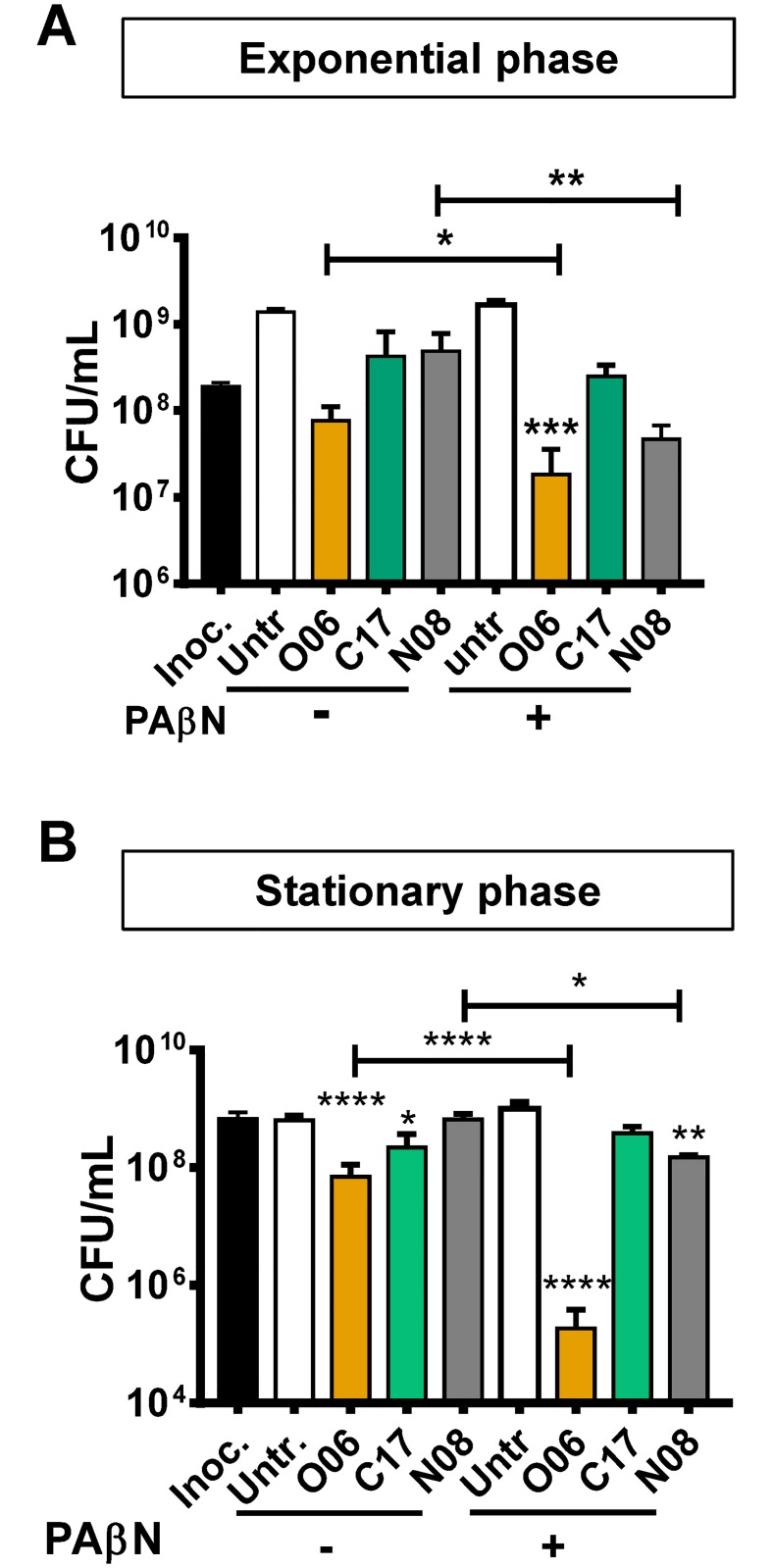
With efflux pump inhibition, compound O06 and N08 significantly decrease the viability of both exponential and stationary phase *K*. *pneumoniae*. CFU/ml was determined after a 4 hr treatment with the indicated compounds at a concentration of 100 μg/mL in the presence or absence of PAβN (40 μg/ml) of A, exponential phase and B, stationary phase *K*. *pneumoniae*. The mean of three independent experimental replicates is displayed. Error bars represent the standard deviation. **** *p* ≤ 0.0001, ** *p* ≤ 0.01, * *p* <0.05, ns *p* > 0.05 compared with the inoculum. Ordinary one-way ANOVA of log transformed data followed by Sidak’s multiple comparison test was used to calculate statistical significance. Compound-treated cultures were compared to the relevant inoculum and +/- PAβN treatments were compared for each compound. Inoc, inoculum, Untr, untreated.

The most effective antibiotics decrease the viability of both actively dividing and stationary phase cells. To compare the activity of each compound in the exponential and stationary phases of growth, LB-grown cultures of a carbapenem-susceptible *K*. *pneumoniae* clinical strain (Kp 233) in each phase of growth were incubated for 4 hours with 100 μg/mL of each compound alone or in combination with PAβN (Fig 5). After 4 hr, colony forming units (CFU) were enumerated. The CFU of untreated exponential phase cultures increased approximately 10-fold during the incubation period, while those of stationary phase cultures remained unchanged. Compound O06, in particular, decreased the viability of both exponential and stationary phase cells. Furthermore, the efflux pump inhibitor PaβN significantly potentiated the killing activity of both compounds O06 and N08 (Fig 5).

As a control, we confirmed that PAβN, by itself, did not decrease the viability of either exponential or stationary phase cells (S4 Fig).

### Compounds O06, C17, and N08 do not significantly alter cell shape

**Fig 6:**
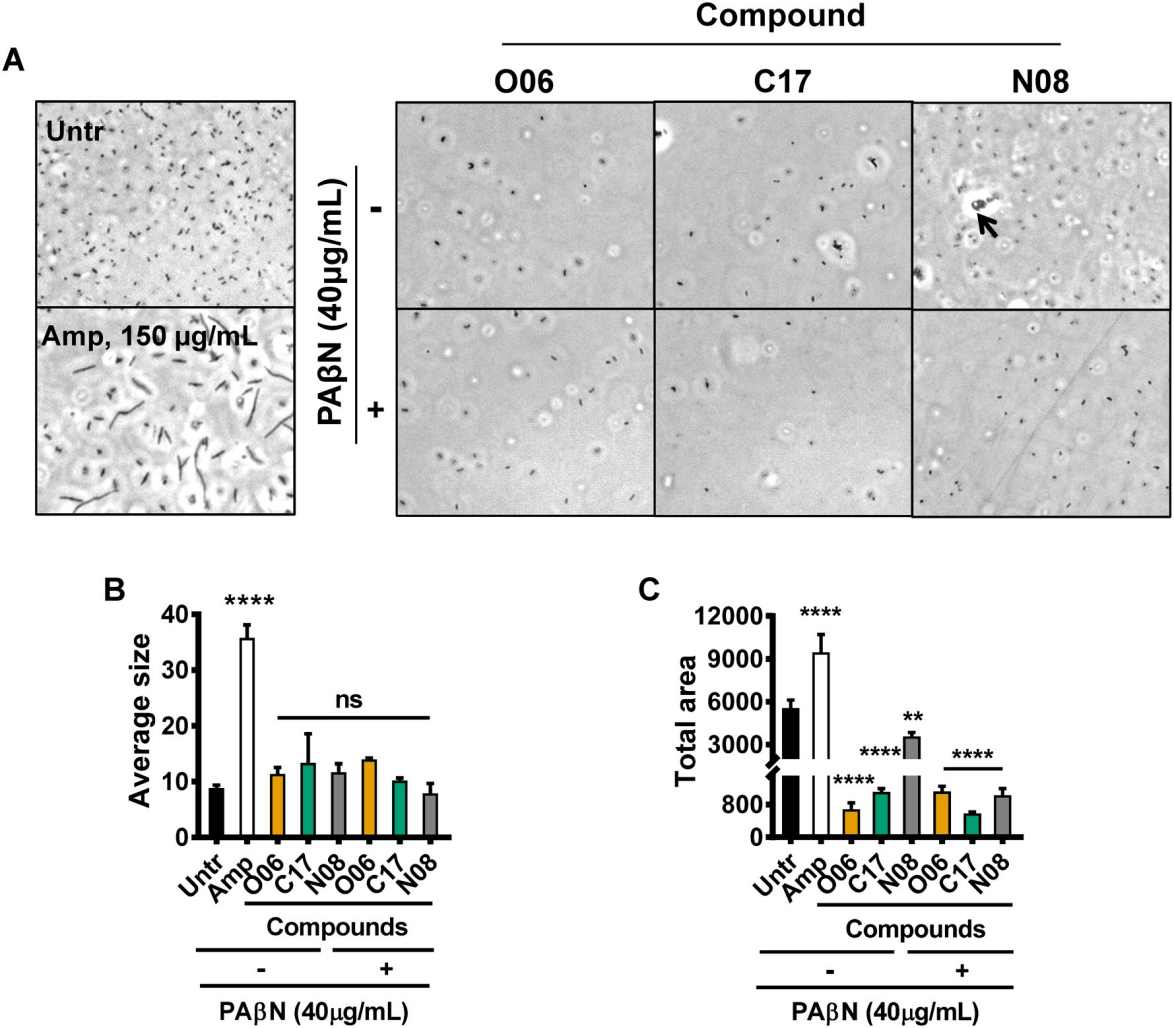
Compounds do not induce changes in cell morphology. Stationary phase cells were sub-cultured into fresh media containing 100 μg/mL of each compound in the presence or absence of the efflux inhibitor PAβN and incubated for 4 hr. A, Phase-contrast microscopy observation of cell morphology after treatment with compounds. Arrow indicates particulate matter identified in cultures containing compound N08. Ampicillin (Amp, 150 μg/mL) is included as a positive control for altered cell morphology of a peptidoglycan-targeting antibiotic. Untr: untreated cells incubated with medium containing DMSO alone. B, Quantification of the average particle size using images obtained in (A). C, Quantification of total area coverage using images obtained in (A). Error bars represent the standard deviation. **** *p* ≤ 0.0001, ** *p* ≤ 0.01, and ns *p* > 0.05 against the untreated (Untr, 0 μg/mL). Ordinary one-way ANOVA followed by Tukey’s multiple comparison test was used to calculate significance.

β-lactam antibiotics, which target peptidoglycan synthesis by associating with penicillin-binding proteins, dramatically alter bacterial cell morphology and are bactericidal [22, 23]. To uncover evidence for a similar mechanism of action, we examined cell morphology by phase contrast microscopy after exposure of a carbapenem-susceptible strain of *K*. *pneumoniae* (Kp233) to our compounds alone or with PAβN. As a positive control, stationary phase cells were exposed to 150 μg/ml of ampicillin or 100 μl/ml of each compound for 4 hr and then visualized by phase contrast microscopy (Fig 6A). The average cell area and cumulative area covered by cells were quantified by morphometric analysis (Fig 6B and 6C). As expected, cells treated with ampicillin formed filaments with significantly increased average particle size as compared with untreated cells. However, while all compounds in combination with PAβN caused a decrease in total area covered by cells consistent with growth inhibition, none of these compounds significantly altered cell size or morphology. These results suggest that none of these compounds acts by a mechanism similar to that of the beta-lactam antibiotics.

We previously hypothesized that compound N08 was quite insoluble in aqueous media. Supporting this, particulate matter was observed in micrographs of bacteria treated with this compound but not those of bacteria treated with C17 or O06 (Fig 6A). We conclude that experiments using medium supplemented with concentrations of N08 close to or exceeding 100 μg/ml may not accurately reflect the concentration of compound in solution.

### Evidence that compound N08 inhibits protein expression

Many effective antimicrobial compounds are protein synthesis inhibitors. We used the carbapenem-susceptible Kp233 strain carrying a plasmid that encodes an unstable variant of green fluorescent protein (GFP) to assess whether these compounds could inhibit GFP expression [24]. As a control, we first confirmed that none of these compounds fluoresced under the conditions of this experiment (S5 Fig).

**Fig 7:**
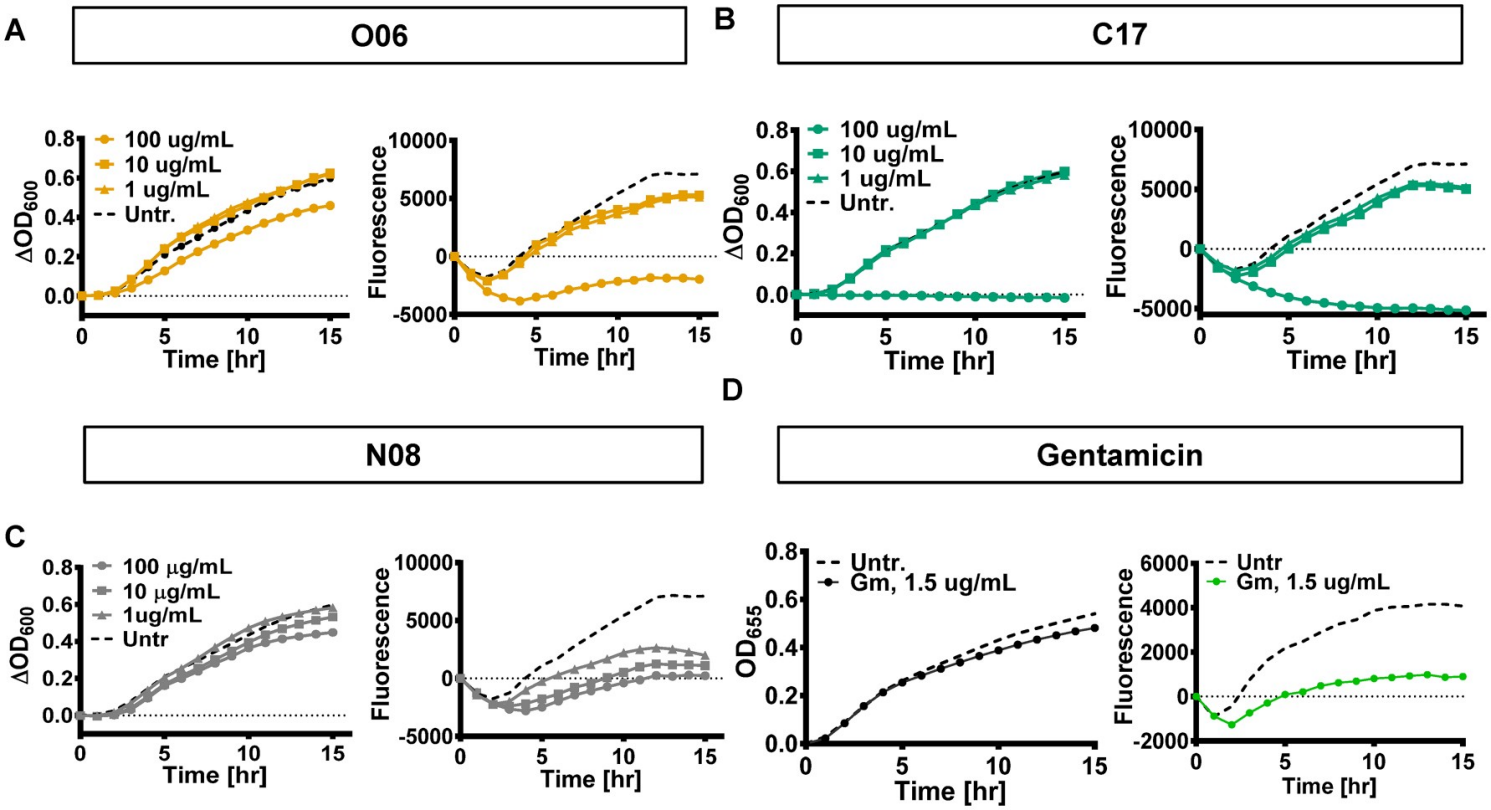
In the presence of compound N08, GFP fluorescence is decreased at concentrations that do not impair growth. Overnight cultures were sub-cultured into fresh LB media containing the indicated concentrations of compound O06, C17, N08, or gentamicin and incubated for 15 hr at 37°C. Fluorescence was measured every 30 min with excitation at 480 nm and emission at 515 nm. Growth was measured by optical density (OD) at 600 or 655 nm. Fluorescence and OD were normalized to the initial level detected at 0 hr to depict change in signal relative to time. Growth and fluorescence in cells treated with A, compound O06 B, compound C17. C, compound N08. and D, gentamicin.

In samples that were not treated with antibiotics or compounds, fluorescence at wavelength 515 nm increased over time as did the OD_600_, corresponding to bacterial growth (Fig 7). Compounds O06 and C17 decreased both bacterial growth and protein synthesis at the highest concentration used. In contrast, low concentrations of compound N08 interfered with GFP fluorescence but not growth. This is consistent with inhibition of protein expression by N08 at the level of transcription, mRNA stability, or translation. However, we cannot rule out a specific effect on GFP that reduces fluorescence.

## Discussion

Here we describe an untargeted, whole cell-based assay designed to identify compounds active against CR-*K*. *pneumoniae*, an organism for which there are few antimicrobial agents. We have used this assay in a HTS of a fungal extract library as well as a chemical library to identify compounds active against a multidrug resistant, carbapenemase-producing *K*. *pneumoniae* strain. Fungal extracts with the greatest activity were shown to contain patulin, a known natural product. Furthermore, we identified three synthetic chemical compounds designated O06, C17, and N08 with antibacterial activity. At lower concentrations, compound O06 functioned as an inhibitor of sugar transport or fermentation but had little impact on cell viability, while at higher concentrations it decreased bacterial viability. In its current form, this compound would not be useful as a human therapeutic, but further investigation is warranted to determine whether it could be of use to researchers seeking to inhibit bacterial sugar metabolism. Compound C17 decreased both sugar metabolism and growth of CR-*K*. *pneumoniae* in the absence of efflux inhibition but did not demonstrate sufficient activity to support further development. In the presence of efflux inhibition, compound N08 was the most highly active against resistant GNRs as well as MRSA. Consistent with our preliminary observations that it acts as an inhibitor of protein expression and possibly synthesis, N08 functioned principally as a bacteriostatic agent. The tendency to aggregate in aqueous media, however, limits its utility.

Patulin, the natural product identified in our screen of fungal extracts, was first discovered as a secondary metabolite of *Penicillium* moulds in 1943 [25]. It was subsequently demonstrated to be a cure for the common cold when used as a nasal wash in the first recorded double-blind, placebo-controlled clinical trial [26, 27]. Its activity against both Gram-negative and Gram-positive bacteria, particularly in biofilms, has been reported [28–31], and there is evidence that patulin functions as an inhibitor of quorum sensing [28, 32], or alternately as an inhibitor of alanine racemase [8]. We did not pursue patulin due to concerns for toxicity [33, 34]. However, this may not preclude its topical use in the treatment of superficial or mucosal infections caused by multi-drug resistant GNR.

Our studies suggest that compound O06 functions principally as an inhibitor of *K*. *pneumoniae* sugar transport or fermentation at lower concentrations. Glucose transport is essential for the replication of *Leishmania Mexicana* promastigotes [35, 36]. This property was exploited in high-throughput screens to identify inhibitors of the *L*. *mexicana* and human hexose transporters LmGT2 and Glut1, respectively [37, 38]. While compound O6 was identified as active in both screens, the results of a secondary screen to directly measure glucose transport are not reported. It is, therefore, possible that O06 functions as a hexose analog that competitively inhibits sugar transport.

Compound N08 has been flagged in diverse screens including one for inhibition of Human tyrosyl-DNA phosphodiesterase 1 (TDP1) and another for inhibition of Lassa virus entry [39–41]. It was previously identified in a primary screen for inhibitors of *S*. *aureus* teichoic acid biosynthesis but was ultimately not found to have this activity in secondary screens [42, 43]. This function would not provide a mechanism for the broad-spectrum activity of N08 as Gram-negative organisms do not synthesize teichoic acid. Compound N08 is also the subject of multiple patent applications disclosing its function as an inhibitor of PAS kinase, potassium channel closure, and TGF-β with possible applications to the treatment of diabetes mellitus, epilepsy, and scarring, respectively [44–46]. This suggests that toxicity to human cells is unlikely to limit its use.

We have demonstrated the utility of our high-throughput screen in identifying compounds active against a highly resistant, carbapenemase-producing strain of *K*. *pneumoniae*. While this approach increases biocontainment risks, our use of an extremely resistant organism in a whole cell screen directly identified compounds active in the face of cell envelope impermeability and efflux. Three compounds identified by the screen, namely patulin, O07 and C17, do not have adequate anti-bacterial activity for further development. Compound N08 has broad spectrum anti-bacterial activity but non-ideal chemical properties. While its propensity to precipitate in aqueous media would limit its direct utility as a therapeutic, this also suggests that its activity was underestimated in our assays. We propose that this compound could serve as a scaffold for a broad-spectrum, highly effective antibacterial agent if solubility and efflux resistance is improved by structural optimization.

## Supporting information

S1 Fig Three fungal extract hits have no cytotoxicity effects on HeLa cells. Extracts F19, F53, and F55 were incubated with HeLa cells for 20 hours. Viability was measured by resazurin reduction and expressed as the percent fluorescence compared to untreated cells.
(PDF)

S2 Fig Inhibition of *K*. *pneumoniae* sugar fermentation and growth by fractions of fungal extract F19. Fractions derived from extract F19 were tested for A, inhibition of fermentation after 7 hr. B, Inhibition of fermentation after 22 hours. and C, inhibition of growth after 22 hours. Gentamicin was used as a positive control. The dashed line indicates the average value of the negative control. The gray area indicates three standard deviations above and below the negative control average.
(PDF)

S3 Fig Inhibition of fermentation by additional compounds. Inhibition of mannose fermentation by additional compounds at 22 hr.
(PDF)

S4 Fig The efflux pump inhibitor phenylalanine-arginine β-naphthylamide (PAβN) does not affect bacterial viability. Colony forming units of *K*. *pneumoniae* harvested at exponential (Exp) or stationary (Sta) phase and incubated for 4 hr in medium containing the efflux pump inhibitor phenylalanine-arginine β-naphthylamide (PAβN, 40 μg/mL) alone. Dotted horizontal line represents the number of colony forming units (CFU) prior to incubation. Error bars represent the standard deviation. A student’s t-test was used to calculate statistical significance. ns not significant.
(PDF)

S5 Fig Synthetic compounds O06, C17, and N08 do not contribute background fluorescence at the excitation and emission spectra of GFP. Compounds diluted into LB medium were incubated for 15 hr at 37°C. Fluorescence (excitation at 480nm and emission at 515nm) and OD_600_ were measured every 30 min withA, Compound O06. B, Compound C17. C, Compound N08.
(PDF)

S1 Table Composition of the defined medium used in HTS. (PDF)

S2 Table Known bioactive compounds identified in the pilot HTS assay. (PDF)
