# Supplementary material for: A high-throughput, whole cell assay to identify compounds active against carbapenem-resistant Klebsiella pneumoniae
Source: PLoS One. 2018 Dec 21;13(12):e0209389. doi: 10.1371/journal.pone.0209389 (PMC6303040; doi:10.1371/journal.pone.0209389)
Supplement: S1 Fig — Extracts F19, F53, and F55 were incubated with HeLa cells for 20 hours. Viability was measured by resazurin reduction and expressed as the percent fluorescence compared to untreated cells. (PDF) [file pone.0209389.s001.pdf]

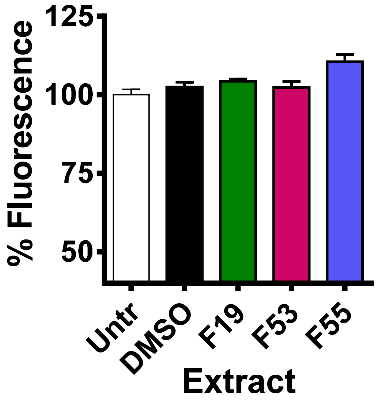

Figure S1. Three fungal extract hits have no cytotoxicity effects on HeLa cells. Extracts F19, F53, and F55 were incubated with HeLa cells for 20 hours. Viability was measured by resazurin reduction and expressed as the percent fluorescence compared to untreated cells.
