# Supplementary material for: A high-throughput, whole cell assay to identify compounds active against carbapenem-resistant Klebsiella pneumoniae
Source: PLoS One. 2018 Dec 21;13(12):e0209389. doi: 10.1371/journal.pone.0209389 (PMC6303040; doi:10.1371/journal.pone.0209389)
Supplement: S2 Fig — Fractions derived from extract F19 were tested for A, inhibition of fermentation after 7 hr. B, Inhibition of fermentation after 22 hours. and C, inhibition of growth after 22 hours. Gentamicin was used as a positive control. The dashed line indicates the average value of the negative control. The gray area indicates three standard deviations above and below the negative control average. (PDF) [file pone.0209389.s002.pdf]

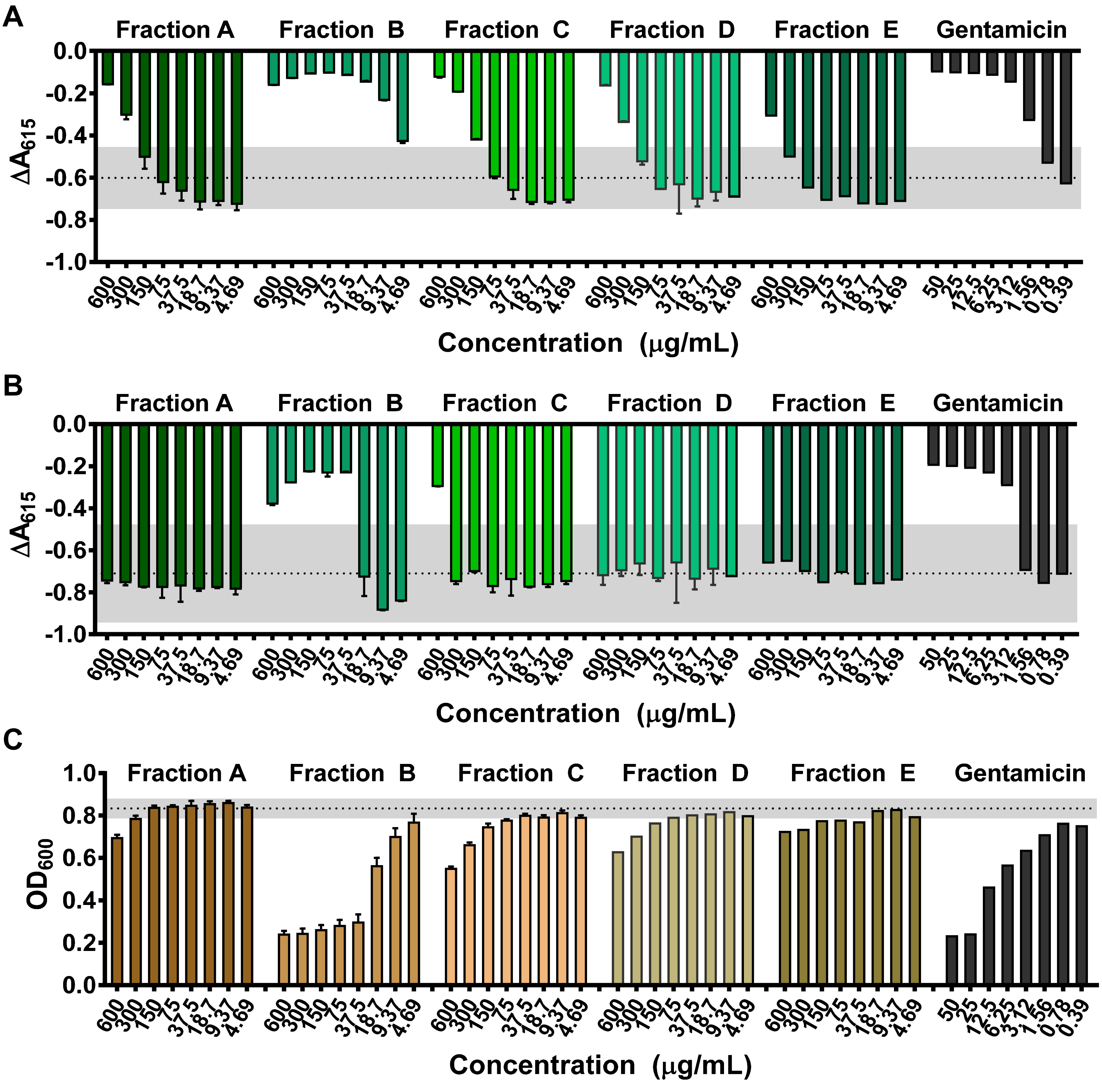

Figure S2. Inhibition of *K. pneumoniae* sugar fermentation and growth by fractions of fungal extract F19. Fractions derived from extract F19 were tested for **A,** inhibition of fermentation after 7 hr. **B,** Inhibition of fermentation after 22 hours. and **C,** inhibition of growth after 22 hours. Gentamicin was used as a positive control. The dashed line indicates the average value of the negative control. The gray area indicates three standard deviations above and below the negative control average.
