# Supplementary material for: A high-throughput, whole cell assay to identify compounds active against carbapenem-resistant Klebsiella pneumoniae
Source: PLoS One. 2018 Dec 21;13(12):e0209389. doi: 10.1371/journal.pone.0209389 (PMC6303040; doi:10.1371/journal.pone.0209389)
Supplement: S3 Fig — Inhibition of mannose fermentation by additional compounds at 22 hr. (PDF) [file pone.0209389.s003.pdf]

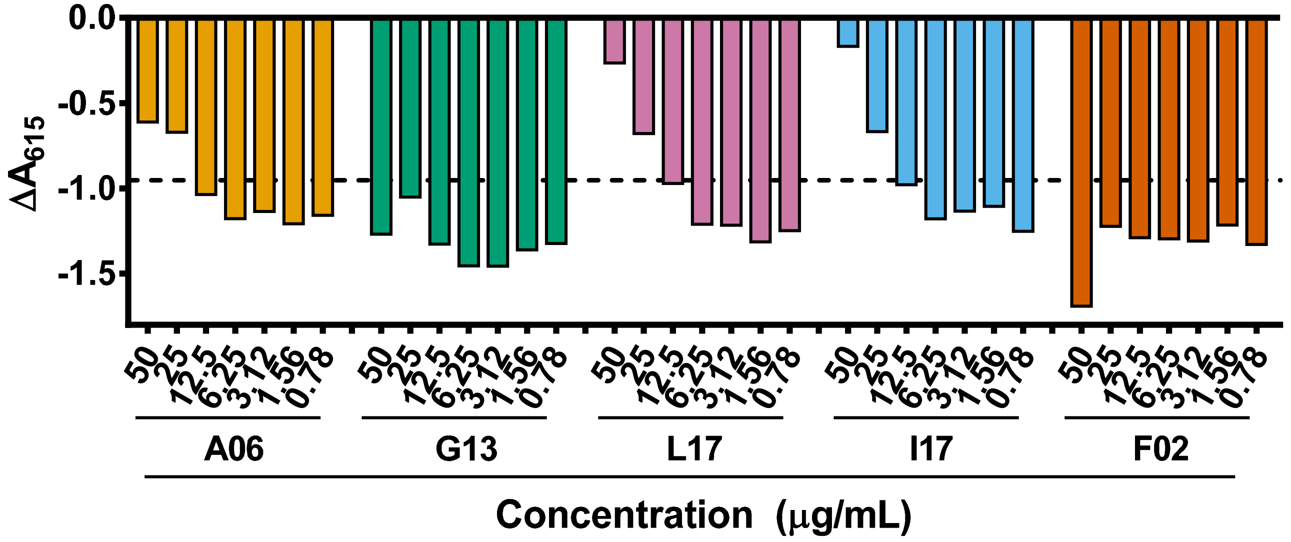

Figure S3. Inhibition of fermentation by additional compounds. Inhibition of mannose fermentation by additional compounds at 22 hr.
