# Supplementary material for: A high-throughput, whole cell assay to identify compounds active against carbapenem-resistant Klebsiella pneumoniae
Source: PLoS One. 2018 Dec 21;13(12):e0209389. doi: 10.1371/journal.pone.0209389 (PMC6303040; doi:10.1371/journal.pone.0209389)
Supplement: S4 Fig — Colony forming units of K. pneumoniae harvested at exponential (Exp) or stationary (Sta) phase and incubated for 4 hr in medium containing the efflux pump inhibitor phenylalanine-arginine β-naphthylamide (PAβN, 40 μg/mL) alone. Dotted horizontal line represents the number of colony forming units (CFU) prior to incubation. Error bars represent the standard deviation. A student’s t-test was used to calculate statistical significance. ns not significant. (PDF) [file pone.0209389.s004.pdf]

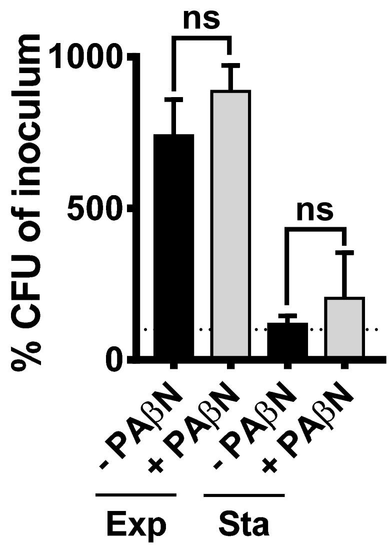

Figure S4. The efflux pump inhibitor phenylalanine-arginine β-naphthylamide (PAβN) does not affect bacterial viability. Colony forming units of *K. pneumoniae* harvested at exponential (Exp) or stationary (Sta) phase and incubated for 4 hr in medium containing the efflux pump inhibitor phenylalanine-arginine β-naphthylamide (PAβN, 40 μg/mL) alone. Dotted horizontal line represents the number of colony forming units (CFU) prior to incubation. Error bars represent the standard deviation. A student's t-test was used to calculate statistical significance. ns not significant.
