# Supplementary material for: A high-throughput, whole cell assay to identify compounds active against carbapenem-resistant Klebsiella pneumoniae
Source: PLoS One. 2018 Dec 21;13(12):e0209389. doi: 10.1371/journal.pone.0209389 (PMC6303040; doi:10.1371/journal.pone.0209389)
Supplement: S5 Fig — Compounds diluted into LB medium were incubated for 15 hr at 37°C. Fluorescence (excitation at 480nm and emission at 515nm) and OD600 were measured every 30 min withA, Compound O06. B, Compound C17. C, Compound N08. (PDF) [file pone.0209389.s005.pdf]

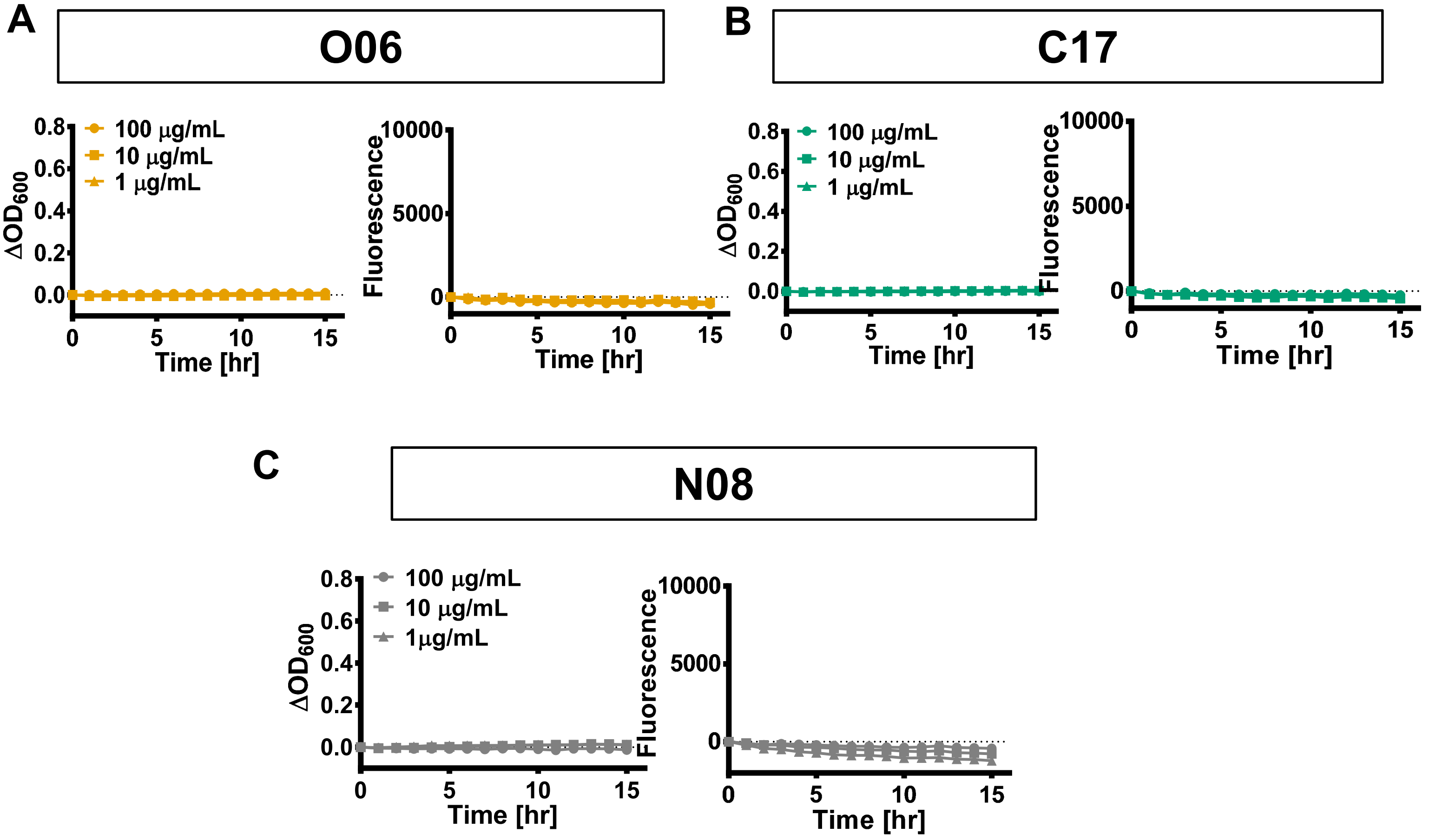

Figure S5. Synthetic compounds O06, C17, and N08 do not contribute background fluorescence at the excitation and emission spectra of GFP. Compounds diluted into LB medium were incubated for 15 hr at 37°C. Fluorescence (excitation at 480nm and emission at 515nm) and OD_{600} were measured every 30 min with **A,** Compound O06. **B,** Compound C17. **C,** Compound N08.
