# Supplementary material for: A high-throughput, whole cell assay to identify compounds active against carbapenem-resistant Klebsiella pneumoniae
Source: PLoS One. 2018 Dec 21;13(12):e0209389. doi: 10.1371/journal.pone.0209389 (PMC6303040; doi:10.1371/journal.pone.0209389)
Supplement: S1 Table — (PDF) [file pone.0209389.s006.pdf]

S1 Table: Composition of the defined medium used in HTS

Component	g/L	mM
Alanine	0.3	3.37
Arginine HCl	0.2	0.95
cysteine	0.02	0.17
glycine	0.13	1.73
Histidine HCl	0.17	0.89
isoleucine	0.3	2.29
leucine	0.5	3.81
lysine	0.5	3.42
methionine	0.1	0.67
phenylalanine	0.2	1.21
serine	0.2	1.9
threonine	0.2	1.68
tyrosine	0.04	0.22
valine	0.4	3.41
KH_2PO_4	0.012g	0.088
K_2HPO_4	0.028	0.16
$(NH_4)_2SO_4$	0.008	0.06
KCl	0.75	10
$CaCl_2 \cdot 2H_2O$	0.05	0.34
$MgSO_4 \cdot 7H_2O$	0.2	0.811
$Fe\ SO_4 \cdot 7H_2O$	0.00017	0.00061
NaCl	5.8	100
mannose	4	22.2
