# Supplementary material for: A high-throughput, whole cell assay to identify compounds active against carbapenem-resistant Klebsiella pneumoniae
Source: PLoS One. 2018 Dec 21;13(12):e0209389. doi: 10.1371/journal.pone.0209389 (PMC6303040; doi:10.1371/journal.pone.0209389)
Supplement: S2 Table — (PDF) [file pone.0209389.s007.pdf]

S2 Table. Known bioactive compounds identified in the pilot HTS assay.

Compound name	Class
**Bioactive plates 3+4 (ICCB 2089, 2090)**	
Rifamycin sv	Antibacterial
Rifampicin	Antibacterial
Minocycline hydrochloride	Antibacterial
Doxycycline hydrochloride	Antibacterial
Clinafloxacin hydrochloride	Antibacterial
Sulfadiazine	Antibacterial
Gemcitabine hydrochloride	Antineoplastic
Doxifluridine	Antineoplastic
5-fluorouracil	Antineoplastic, anti-metabolite
Floxuridine	Antineoplastic, anti-metabolite
Auranofin	Anti-inflammatory
